# Our Annual Income as an Element in Dental Progress

**Published:** 1905-07-15

**Authors:** B. J. Cigrand


### OUR ANNUAL INCOME AS AN ELEMENT IN DENTAL PROGRESS.

BY B. J. CIGRAND, M.S., D.D.S.

Read before the Southwestern Michigan Dental Society, April 12, 1905.

It is the general impression that in our conventions the subjects as pertain to the technic and science of dentistry alone beget or foster progress, that the advancement of our profession depends entirely on phases of our tasks as enter into operations on the dental organs, their adjacent parts in the oral cavity.

It is the purpose of this paper to bring to your attention a feature in dental progress which has not attracted in this sense any consideration.

These gatherings are convened for the express purpose of advancing the art and science of dental surgery, and the primal object is to be of greater service to suffering humanity, hence the inference that only such subjects as pertain to the technic or science has any bearing on dental progress. This narrow and generally accepted view has deterred our profession from commanding the cordial consideration to which it by right is entitled.

The kindred arts, sciences, callings and professions are taking advantage of the so-called spirit of the times, and are shaping their destines to fit the colateral circumstances of unversal—not special progress.

I assure you in the proem of this paper that I shall not take a selfish view of this perplexing question, but shall aim to treat it in a broad and liberal scope. I hope to convince you that all I say shall tend to enlighten the public, serve and benefit the public, and only in an indirect sense accrue to us better financial results.

Our profession has devoted considerable attention to organizations, associations, societies, clubs and fraternities, calculated to advance the purely scientific side of dentistry. Let us presume that we are a unit as to the etiology of dental caries, that we fully comprehend the causation of pyorrhea, that our knowledge is complete concerning root-canal dressings and that the underlying principles of crown work are fully known. Hence let us dispel from our minds all such subject matter as pertains to the technic and practice of dentistry and consume the time with a theme fully meriting our closest attention. This paper hopes to express in an emphatic manner the necessity of closer fraternalism on the interesting subject of professional remuneration.

This is an age of centralization; the trend of the times is toward combining forces and uniting energies of a similar character. At no period of civilization has there been expressed so pronounced a purpose of affiliation. Institutions of industry and commerce have recognized the good results growing out of mutual concern. The individuals, too, discern the benefits of such organizations and hence union of effort has resulted in general uplifting of condition. In fact, this disposition on the part of both, corporate and individual enterprise, has brought about circumstances which control the growth and progress of every known human effort, our own profession being influenced in countless ways. This spirit of association has ripened to such a degree that union and existence have, in many vocations, become synonymous terms.

If we, as a profession, aspire to command public esteem as professional and public servants, we have much yet to accomplish—other than mere technical and ethical proficiencies and standards. Our culture and our general behavior in the society and community in which we live will contribute most pronouncedly towards inducing the public to pay us higher financial and social tribute.

The individuai, his early training resulting in a clear, positive and admirable character, is an equation we must not lose sight of. Nature may have endowed him with a force in both, physical and mental aspects, which readily places him in a favorable position to command the debt of respect, accompanied with a willingness to pay a high compensation; again he may have, by close application, improved his heritage and developed such elements as he felt essential for a professional success. His pursuit of knowledge which gradually converted his love of art into admiration of the beautiful and his eagerness to learn the science of known, soon lead him to a study of the realms of the unknown, unconsciously builded within his own body and mind a gentle, refined honest, forceful and proficient professional gentleman. With this acquired manhood he becomes a power, he gathers within him an undescribable control of circumstances, kindred to his training, he gains prestige and wins the favor of the entire community and in this chronicle of apparent success lies the harvest of real success, for nothing succeeds like success.

The profession to-day is ready for advance in standards of compensation, we need not delay longer; we have attained to a position in the civic scheme where we are necessary. What next shall we do? The general surgeons of to-day are awakening to the importance of our professional service. Only recently Dr. D. A. K. Steele, the consulting surgeon of the College of Dentistry University of Illinois, in a conversation assured me of the importance of normal conditions in the mouth in patients awaiting operations on the alimentary tract. He said it was essential to prevent the presence of pathogenic energy, at surgical wounds, ulcerated or diseased oral tissues or disturbed dental organs, which harbored pus contributed this deadly element to the food and the latter, freighted with these pathogenic organisms, encroaching upon the new wound, produced inflamation and the consequent irritation and not infrequently resulting in death to patient. Hence he has made it the initial step in his examinations to determine that no such abnormal conditions exists in the mouth, thus avoiding threatening complications in internal surgery involving the alimentary tract. Stating that he considers an examination of the oral cavity was essential to ultimate success.

As further evidence to the high appreciation accorded our calling these trans-atlantic words will be welcome: "Joseph Kidd, M. D., an eminent Enlgish physician and specialist, deals with the cause and cure of appendicitis in a manner and from a point of view which make his observaions of exceeding value and interest to the lay reader. Dr. Kidd has had fifty years of extensive practice in London, and it is his testimony that appendicitis was very infrequent until about twenty years ago. As for the causes of the disease, he attributes it often to a chill. Hurried eating and imperfect mastication, he says, are very potent causes of appendicitis.

Masticate well, eat slowly, do not swallow any food that is not perfectly softened by the teeth. Even salads, fruit, nuts, almonds, raisins, may be taken freely if well masticated. In the haste and bustle of city life it is better to take half a meal well masticated than to bolt the whole in a hurry.

All this contributes to the importance of dentistry and augments our services. If other specialists of the mother calling can exact high fees for their services, who in this audience, or who of the masses can assign good causes for our failure to deserve better considerations. The financial gain would redound to the welfare of the commonwealth and the public generally.

Statistics will prove that the dentist more than any medical specialist must expend his monies liberally if he hopes to keep in touch with the rapid onward strides of his calling; he more than any of his co-laborers in the field of medicine or the art of surgery must be in actual communion with his fellow practitioner. This at first though may not be understood and will quite likely be underestimated.

Our vocation deals pronouncedly with the mechanical with the digital, with the manual. With us the fingers must work in concert with the mind. We must idealize, then with our hand realize. The importance of associating with our professional brothers, and coming in contact with their material successes is essential. The benefit of gatherings, such as these, are fully understood by the progressive, ambitious and earnest dentist. He comes to contribute and to receive; he is here to see how various appliances are adapted, how certain proceedures are instituted, how the mechanism is constructed and what method is applied in obtaining results in harmony with the laws of mastication and basic rules of anesthesia. You may attempt to tell him this by the means of the pen or type, but he will lack the element of proceedure and hence he will delay attempting a similar task. You cannot transfer manipulative steps nor digital movements, nor material changes with any degree of certainty by word pictures. All vocations or tasks with manual skill as their basis are best taught by the eye, hence the failure of teaching the art of violin or painting or sculpture by correspondence. The operator must exist in your eye; he is the pattern, his every movement is a symbol of product, and the contact and communion with him is the element which creates in you the spirit of progress and accomplishment. The eye and the hand are inseparably associated with dentistry. Hence dentists must expend large sums of money to attend the various gatherings of dentists, local, state and inter-state. This means time from the office and labor and often great tedium to himself.

How different with the attorney, the pharmacist, the theologian and even the physician, who can be in touch with the actual progress of their respective professions through the medium of their journals and magazines and reports. Immediate and personal contact is not necessary since the progress and advancement of these professions is devoid of digital dexterity.

The public is the immediate benefactor of this gathering—we are assembled to evolve methods calculated to relieve pain, institute improved appliances and deduce proceedures calculated to inaugurate greater comfort and bequeath health and longevity to an infeebled humanity. As the populace enhance our compensation, we endow them with greater blessings.

Operations which demand skill and conscientious attention, as the administration of anesthetics, where the practitioner practically takes life within his charge, the fee is so insignificant that I will not dignify the price paid to pronounce it a fee, compensation, or remuneration, but stamp the charge by the commercial word price, which has within its meaning the unstable definition of a fluctuating bargain-counter symbol. Could you dream of the lawyer, oculist, surgeon or physician assuming the responsibility of human life at the paltry sum that it would require four cases to net one dollar. Such a disposition on the part of dental practitioners holds our calling on the low level of a trade. Then we have the subject of root-canal fillings, those distressing, tortuous canals where you waste life force, exhaust your nervous energy and bring to your entire being, mental and physical, a tedium not yet clearly described in our dental vocabulary. I sometimes think this operation oft brings a peculiar tired feeling not yet properly named.

This trying, exacting and painstaking dental operation, for the most part, does not receive the consideration of meriting any charge.

The surgeon often performs operations purely within the dental province, yet receives financial consideration decidedly in excess of what the dental practitioner might hope to obtain.

Wherein is our equity in such a procedure? Why can the physician and surgeon exact fees for services we have every possible license to perform and we submit a bill reduced by a large devisor?

When we analize the previous training and general career of the surgeon or physician, we cannot comprehend the causes for such flagrant discrimination in adjustment of equivalent surgical services. We devote sixteen years to our training—so do they. The percentage of dentists who hold the M. D. degree far exceeds the physicians who hold the degree of D. D. S. As a profession we are the best qualified and organized—why hesitate to charge?

The question of tenure of service may not appeal to you as being of any importance in registering our equity in the matter of remuneration, but careful study will convince you that the duration of dental service is considerably shorter than the other professions. Our hours of work are considerably limited, and all we earn comes from the chair. In short, we are not able to practice at such an age as the physician and lawyer, we have as our harvest time a period of about fifty years, granting that the dental student at the age of twenty-one receives his degree, at the age of seventy-one he will have attained to that mile-stone of life where he can no longer hope to merit the confidence of new clients. Your age will have incapacitated you for the finer technic your eyesight likely sustained injuries from the strain of our specialty while the constant caution has provoked a variety of nervous disturbances and the peculiar pose of the dentist at the chair, yielding little exercise, inaugurating some weakenings, hence the years for actual practice are placed at a high figure at seventy-one. Dr. C. Edmund Kells, Jr., of New Orleans, wrote: "The years of advantageous practice of a dentist are comparatively few, with the lawyers and physicians each succeeding year but adds to his experience and usefulness; but with the dentist it is quite different, for at the age when either of the others would be in the prime of his profession, the dentist is on the decline."

This we all accept as the plain truth, hence the dentist must he better paid, since in his years of decline and retirement he will require the means earned while in the days of vigor.

The verdict is that two causes induce the high mortality rate. First, position at the chair and confinement in small offices. Second, likelihood of infection from consumptive patients. The percentage of dentists who succumb to tuberculosis is placed at 11 percent, while lawyers show as low as 8 percent and physicians barely 7 percent. Physicians suffer least from nervous ailments, while lawyers register higher and dentists the maximum. These figures go to demonstrate that our vocation demands fewer hours, longer vacations, better salaries to lengthen life as compared with the sister callings. Rest is but another term for rejuvination, and this in turn means vigor, and without vigor you cannot accomplish good results in dentistry and naturally cannot demand a corresponding good remuneration. The element of health enters into the equation of salary and fees in a most emphatic manner. The most dreadful calamity that can befall one of our profession is to allow his practice to make a slave of him, to toil and labor without recreation, to so highly esteem the pleasure of operating for your patients as to deny yourself the necessary breathing spells and opportunities for nervous re-enforcements. A most troublesome point is the method of arriving at the judgment of the fee. Many dentists charge by the "clock system," others by the "service rendered system". I am inclined to the belief that the operation when complete should merit such a fee as is in accord with the material used, energy employed, disposition of patient, and general standing of patron, regardless of what the clock was doing in the meantime. The clock system has so many objectionable features that I have long since taken the clock from the eyes of my patient. When you follow the clock you feel so completely at the mercy of your patient that it annoys you to answer the 'phone; distresses you to welcome a new-comer, and grieves you to shake the hand of a friend, every moment of the specified time belongs to your patient, you are a *serf;* when you leave the chair to attend to these essential interruptions, you must stop the professional clock, much as a time-keeper at a foot-ball game stops his watch to allow for recuperations and injuries. I have discarded the time system, I despise the tread mill, I love to live.

I quite agree with Dr. McKellops, who once told me this about fees: "I never allow a patient to snap a watch at me as an inference to hurry and keep down the price. I am not racing with either sun or moon when I am inserting a gold filling."

It is true your patients will compare notes and get acquainted with your prices, but we must at some time convince the public of the variety of circumstances which control the charges of any given operation. I contend that scarcely any two operations, even in the same mouth, are precisely similar, hence I have fought the idea that Richmond crowns, dentures, fillings, treatments or even extractions, should have a standard price. We do not deal in commodities, we do not aim to typify in our reproductions we are controlled by living organisms, subject to the great and divine law of molecular change, and correspondence; hence our dominion admits of a variety of charges for what appears to be the same operation or construction.

The Chicago *Daily News* has this splendid article on the matter under consideration:

"Municipal dentsits are appointed and paid by many of the large towns and cities of Germany, says the New York Tribune. In Strasburg, for example, 2,666 children were examined last year, 699 teeth were filled and 2,912 extracted.

The method of work is simple. The teacher brings his class to the dentist, who examines each mouth quickly and marks on a card each child has brought whether treatment is necessary. If so, the child must come again on a Saturday. Russia is also joining in this movement and has already fitted up nine such institutions in St. Petersburg.

And why not, or rather, why so late in coming, one might ask. If it is true that, generally speaking, good teeth are necessary to good health and long life, and if, also, a large and growing proportion of citizens have not good teeth, then it follows that the fact is one of public concern. Is it not, for instance, of as much importance to the community that workmen should have good masticating and digesting powers as that there should be $20,000,000 city halls, public parks, expositions, etc.? This little, or large, realization of preventive medicine has so far got into our American minds that we have ordered the soldier's teeth to be attended to and his governmental service by so much enchanced. But the soldier is at last paid by the civil worker and as to his teeth and service we are entirely indifferent."

It is a part of a nation's duty to see to it that her citizens are strong, healthy and vigorous. Her defense depends on the physical perfection of her soldiery, and can she expect stanch and endurant cadets or volunteers whose physical being has been nursed and developed by unwholesome and impure substances?

All governments at present are keenly alive to the interests of the commercial and material things, hence this craze for oceanic speed and military art. The United States annually spends millions of dollars in the department of agriculture in the hope of arresting the disease of swine, cattle and sheep. The government provides the scientists with the best of lenses to discover and decipher, the bacteria and agents of destruction to the animal and vegetable kingdom, but the human family is left to the individual enterprise and disease, hovers at every door step in the form of consumption, dyspepsia and pyorrhea allowing a death rate of a most alarming figure. The government could well afford to publish fewer books on cattle and swine bugology and devote a portion of this enormous sum to the redemption of the citizen health.

The general government appropriates funds for the medical advancement and research, while the dental is relegated to individual effort and society endowment and all present know what a hardship this investigation is entailing to those few of our profession who painstaking labor and eagerly forego pleasures and welcome self sacrifice in the interest of humanity, all without compensation and in the majority of instances inducing loss of practice and forfeiting their general health.

If the general public knew the true status of affairs as pertains to our present meager means for prosecuting investigations into realms of direct concern to human life, if we could so organize our profession as to impress the legislators or even influence the civic authorities of the essentials of public funds for public good, we would have accomplished an invaluable good and done more for the advancement of dentistry as founded on true arts and real science than can possibly be foretold or estimated.

### DISCUSSION.

Dr. F. H. Codding: This paper is difficult to discuss because the essayist has put the matter so strongly and broadly. There is no question in my mind about the question of compensation for dental services, and there can be none in the mind of the faithful and conscientious practitioner. Some patients appreciate our efforts and do their best to compensate us, while a large number are inclined to traffic about a matter of such vital concern. It would be well if we could live without fees, but we can't, and the people whom we serve must compensate us. Not only are we compelled to provide for our present needs, but we should also anticipate the time when we can no longer accomplish so much as in the days of strength capacity. One great fault with dentists is that they do not collect their fees as promptly as they should. I do not boast when I tell you that I collect 98 percent of all the business I do. Some people think it smart to beat the dentist out of his fees, and the dentists practicing in a community ought to have a black list for each others benefit. In recent years all kinds of expenses have materially appreciated except for professional services. It would be more profitable for us to go fishing than to work for people who will not pay their fees. If we keep abreast of the times it not only costs money, but effort. And in these days of required cleanliness we have greater demands made on us than our predecessors and we should have increased compensation. We can't base our fees on cost of material and living expenses, the wear and tear of mind and body should be important elements for consideration. There is a certain amount of charity or benevolent work to be done in every practice, but it should be done exclusively on that basis. Where patients can pay, the fee should correspond with the service, and no fee that has been earned should remain uncollected.

Dr. S. M. Fowler: I am heartily in favor of the essayists object and also the gentleman who has just discussed the paper. He is surely a good business man to collect so nearly all he earns. In my city, for instance, the gold crown has gradually gone down the fee toboggan slide, until it has struck the four dollar mark, which I am free to confess is all that it is worth in most cases. Now how are you going to educate 98 percent of these people out of the notion that a four dollar crown is the proper service for them to have?

Dr. Rix: Dr. Codding was in business with me at one time, and I can substantiate his statement that he is a good collector. When we dissolved partnership he turned over to me a lot of accounts and I have them still. In regard to a fixed price for services, I think we have made a mistake. No two services are so similar as to make it practicable to adjust the fee in advance of the service. Render the service and then exact a fee that compensates. Professional men are, as a rule, poor business men. I have saved some money from time to time, but invariably some one who thinks he can handle my funds better than I, has come along with a great scheme and my money goes; I know not how or where. It is only the money that I have invested in professional affairs that I feel that I still own.

Dr. E. A. Honey: The number of dentists in this country has practically doubled in the last twenty years and the majority of them, at least, are busy and seem to be making a living. This would indicate that people are appreciating more and more the value of the dentist. It is also significant that people are demanding those services which require the higher degree of skill, and we find that every busy dentist to-day is equiping himself with the facilities for rendering this quality of service. It seems reasonable that the more exacting the service the better the fee should be, and it will be strange, indeed, if we should attain a high degree of skill and not be ready to demand a corresponding fee. If we are compelled to render better service we should be entitled to better fees.

Dr. George W. Cook: I want to express my admiration for Dr. Codding as an ideal business dentist. He talks as though he was one of those fellows you read about, who shoot their patients if they don't pay their bills promptly. I have some that deserve to be shot. I have worked hours on a nervous patient endeavoring to extract a pulp nodule and exhausted my mental as well as physical strength more than a surgeon would on a case of apendicitis, and yet I have not been able to collect a fee that would, in any sense, compare with the surgeons.

I started in to educate myself for surgery, but concluded there was more opportunity for the use of the natural bent to mechanics, which I have in dentistry than surgery. The surgeon may be a little more broadly cultured, but he is not so skillful a man as the dentist, and is not called upon to take the initiative so often as the dentist. The general public and some physicians do not appreciate the high order of skill required in the practice of modern dentistry. We must make our impression on the public by rendering a higher order of service and exacting proportionate fees for it, and at the same time deserving it by increasing our special as well as our general culture.

Dr. A. S. Rowly: Dr. Cigrand's paper is timely. He has attended every meeting of this society and always brings some good thought. It is a good plan to think well of ourselves, because we are then in a proper frame of mind to make other think well of us too. If we can only make people believe that apendicitis has its origin in bad teeth, we shall have little trouble in making a favorable impression on the public. It would be well if we could realize more than we do the important relations existing between the mouth and the other vital organs of the body. We should then have so much respect for the teeth that we would save more than we do now.

Dr. A. C. Runyan: Our profession is yet a young one, and the public knows that. It has, however, given us a fair measure of credit and I have no doubt but we shall some of us see the day when dentistry will take its place alongside of other specialties of modern medicine. I see no reason for complaining of the position the public has accorded us. The greatest discrediting our profession has ever received has come from within our own ranks. When we have learned to live with our conferes in the professional spirit, we shall have no cause to enter the complaint that Dr. Fowler makes of the four dollar crown. When we shall assume the dignity of a profession and practice it worthily, the people will be very glad to accord us not only place, but dignified fees for services.

Dr. J. N. Thompson: We do too much work for no fees. Dentists, as a rule, are rediculously imposed upon in the treatment of teeth. These services are usually prolonged of necessity, and when we charge for the time consumed, it makes a large fee for work which does not show to the patient. This is largely due to the fact that people are accustomed to pay small fees for this kind of a service. It is high time that our colleges and writers in general should take up this subject and put it on a rational basis. Many young college men do not know what to charge for service as the only experience they have had was gained in the college clinic.

Dr. Cigrand: It is estimated that not more than 10 percent of the people have other dental services than the extraction of teeth and such trifling operations. The other 90 percent can be and should be brought under the influence of our ministry. As a business proposition we can get better fees for our services if we ask and earn them. When you make out a bill do it in a professional way, and not itemize as your butcher does. If your patients can trust you with their lives they certainly should trust you to charge them a fair fee. Patients get an idea that you are selling your service at a given rate when you itemize an account, or that you are selling the materials you use. Another thing, don't charge by the clock. An hour given to one patient is worth two or three times as much as when given to another who behaves himself and gives you a fair chance to render a proper service. If you charge by the clock the patient will be tempted to call time on you. When I am serving a patient, I don't want to be bothered by a clock. I want to do my work right or not at all. Patients can have no just idea of what we are doing or how we are doing it, they must trust us and we should never abuse a patients confidence. A good motto is: "Run your practice, dont let it run you." We can learn a lot by keeping close to the medical practitioners. They belong to an older profession and their methods are very well established, and the nearer we can approach their method the more good will come to us.

